# Oil Removal in Prewet Calcite: Active Versus Inactive Ions Investigated by a Fourier Transform Infrared and X-ray Photoelectron Spectroscopy Study

**DOI:** 10.1021/acs.langmuir.5c00286

**Published:** 2025-04-07

**Authors:** Jesana M. Loreto, Emilia Annese, L. G. Pedroni, Fernando Stavale

**Affiliations:** † Brazilian Center for Research in Physics, Rio de Janeiro 22290-180, Brazil; ‡ Center “Leopoldo Américo Miguez de Mello” for Research and Development (CENPES), Petrobras S.A., Av. Horácio Macedo, 950 - Cidade Universitária, Rio de Janeiro 21941-915, Brazil; § Chemistry Department, Pontifical Catholic University of Rio de Janeiro, Rua Marquês de São Vicente, 225, Rio de Janeiro 22451-900, Brazil; ∥ Instituto de Física, Universidade do Estado do Rio de Janeiro UERJ, Rua São Francisco Xavier, 524, Rio de Janeiro 20550-013, Brazil

## Abstract

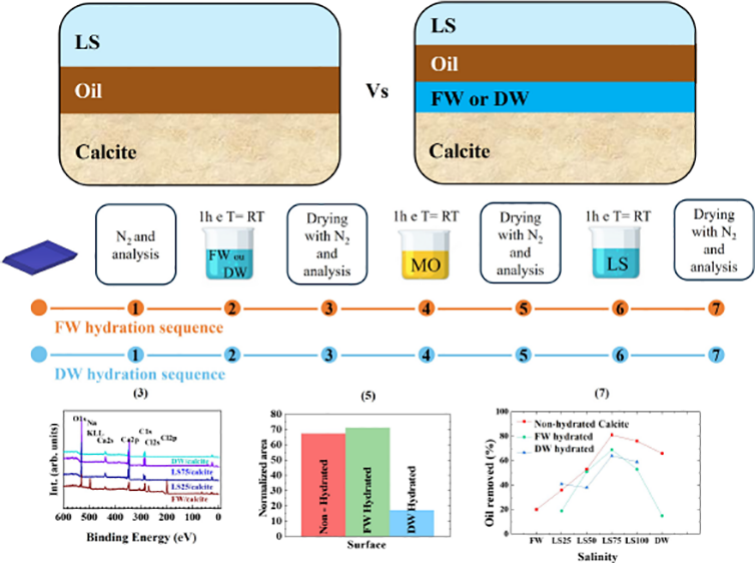

In presalt reservoirs, natural rocks interact simultaneously with formation water (FW) and mineral oil. The prehydrated calcites with FW and demineralized water (DW) are suitable model systems to investigate oil adsorption (removal) on (from) their surfaces by Fourier transform infrared spectroscopy and X-ray photoelectron spectroscopy. Preliminary characterization of the chemical composition of fresh cleaved calcite conditioned directly with low salinity (LS) waters, FW, and DW indicates that calcite undergoes (i) surface dissolution once in contact with DW and diluted LS water (LS100) as testified by the split of ν_3_ vibration bands of the CO_3_^2–^ group and (ii) partial chemical modification of calcite e through Mg incorporation, water anchoring at the surface, and salt deposit formation at the calcite/FW interface. Pristine and (FW, DW) prewet calcites were conditioned with Nujol, and an overall larger CH_2_ and CH_3_ vibration band intensity of Nujol was observed on the FW prehydrated surface than calcite/DW/oil and calcite/oil interfaces. The final conditioning of calcite/oil, calcite/FW/oil, and calcite/DW/oil with LS water ended up with greater oil removal for a saline solution of 0.2 mol/L ion strength, independent of the prehydration. Our results indicate that altered calcite chemical composition or dissolution affects subsequent oil adsorption and removal, and, therefore, there is a competitive role between brine ion strength and oil and surface compositions in the enhanced oil recovery process using LS water.

## Introduction

Enhanced oil recovery (EOR) is a strategic approach in the petroleum industry that aims to increase the efficiency of oil production by maximizing its yield and economic viability since conventional recovery methods have already reached their limits. EOR methods are based on the modification of the physicochemical properties of fluids and their interfaces in the reservoir.^1^ Among them, injection of low salinity water (LS) was demonstrated to be promising for oil recovery, minimizing the environmental impact.^2−4^ Salinity quantifies water ion composition; therefore, LS indicates water with a diluted ion concentration than formation water (FW) found in the reservoir.^4^ LS water interacts with rock and induces modification of the oil/rock interface, such as a decrease of interfacial tension and wettability alteration.^5−7^ Its efficiency depends on the chemical characteristics of the rock (e.g., calcite vs sandstone) and the oil and the injected water composition. The presalt of South American reservoirs is mainly made of calcite,^8^ and therefore, we focused on the LS water interacting with the calcite surface. The crude oil in the natural reservoir can be a mixture of molecules such as carboxylic molecules, fatty acids, alkanes, toluenes, and asphaltenes and impurities such as sulfur, nitrogen, and metals.^9^ Its composition is strictly related to the geographical source, and linear hydrocarbons may vary from 70 to 28%, going from Arabian to South American reservoirs.^9^ Up to now, mixtures of light and heavy hydrocarbons have been used as model oils to investigate the EOR process.^10−14^ However, the role of specific linear hydrocarbons in the wettability alteration of the rock is still of interest for the scientific community. Moreover, due to the oxide nature of minerals in the reservoir, a possible acid-basic reaction at their surfaces could not a priori be ruled out to induce polarity to any polar molecule.^15^ Among the linear hydrocarbons, Nujol was selected as the suitable one for the current work. Its adsorption on rocks can be driven by either van der Waals or electrostatic forces.^16−20^ Previous studies showed that Nujol adsorbs on a fresh cleaved calcite surface mainly by van der Waals interaction.^21^ However, in a real reservoir, rock is surrounded by both FW water and crude oil, and either the water ions or the oil absorbs it. Calcite surface hydration prior to oil and water conditioning may represent a way to artificially reproduce the setting up of the oil reservoir environment in a laboratory. In fact, a significant modification of oil–rock interactions may occur when prior hydration is accounted for.

Therefore, the role of single ion or saline solution in the oil removal process was investigated on a prewet calcite surface.^22,23^ In the prewet process, some of the ions (such as Ca^2+^, Mg^2+^, and SO_4_^2–^) of saline solution are expected to be retained on the surface as a thin layer,^23^ and their presence may promote surface wetting alteration and Nujol removal enhancement.^22,24^ Among those ions, Ca^2+^, Mg^2+^, and SO4^2–^ are expected to favor oil removal, although even Na^+^ and Cl^–^ can be active ions at low ionic strength.^23^ These ions may exchange and replace one of the rock surface or contribute to the ion double-layer formation at the calcite surface.^25,26^ In this work, FW and demineralized water (DW) were used for prior calcite hydration. FW is rich in salts and reflects the in situ conditions of the reservoir, whereas DW is ion-free; therefore, both treatments are expected to modify the ion distribution at the surface that could either favor or hinder oil removal. Each step of the sample treatment (prewet, oil immersion, and LS conditioning) was monitored by attenuated total reflectance Fourier transform infrared (ATR-FTIR) recording OH, calcite, and Nujol vibration bands. To evaluate the Nujol amount on the calcite surface, we used a reliable procedure: all oil-wet surfaces were produced using the same methodology (as in refs (21 and 27)). After each treatment, the Nujol left over on the surface was detected using CH_2_ and CH_3_ vibration bands and quantified by monitoring their relative intensity variation.

Moreover, representative interfaces were investigated by X-ray photoelectron spectroscopy (XPS) to evaluate the element surface composition and oil left on it. XPS is chemical and site-selective, i.e., able to probe elements in different chemical environments (inclusion vs deposit formation). The enhancement of element concentration at the surface may affect the oil adsorption (providing different anchoring sites) or its removal. In fact, oil-wet dolomite response to smart water is less effective than that of oil-wet calcite, and these natural rocks differ only by the partial occupation of the Ca site with Mg.^28^

## Experimental Section

### Water Preparation

The FW was prepared by dissolving NaCl, CaCl_2_·2H_2_O, MgCl_2_·6H_2_O, KCl, NaHCO_3_, and Na_2_SO_4_ in DW under magnetic stirring to ensure the homogeneity and complete dissolution of the salts. To reproduce the salinity of South American reservoirs, FW salinity was set to 238,000 TDS (mg/L) and established by salt weight.^21,29,30^ LS water was obtained by diluting FW in DW according to the following ratios: 1:25 (LS25), 1:50 (LS50), 1:75 (LS75), and 1:100 (LS100). After preparation, the brine pH was adjusted to ∼7 using 0.1 M NaOH or HCl. More details about the water preparation can be found in ref (21).

### Sample Conditioning Procedure

**Figure 1:**
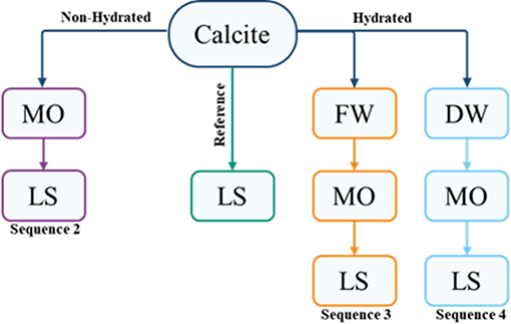
Sequences of interactions carried out in calcite single crystals.

Natural calcite single crystals were supplied by LEGEP MINERAÇÃO Ltd. Their surface was prepared by cleaving the rhombohedral-shaped samples along their (104) plane.^21^ The fresh cleaved crystals were subjected to conditioning sequences with and without prior hydration of the surface (Figure 1): in the reference green sequence calcite is conditioned with with FW, DW, and LS waters; in purple sequence it was treated with Nujol and subsequently in the LS waters (refs (21 and 30)); in orange and blue sequences calcites were pre-hydrated with FW and DW and afterward is conditioned with Nujol and LS waters. All the treatments were carried out by immersing the crystal in the liquid (FW, DW, LS water, and Nujol) for 1 h at room temperature. The samples were gently dried by a N_2_ flow.

### Sample Conditioning Procedure

ATR measurements were conducted using a vacuum FTIR spectrometer model VERTEX v70 (Bruker). The spectra were obtained in the 4000–400 cm^–1^ range with an average of 64 scans and a resolution of 4 cm^–1^ and analyzed using OPUS software. All spectra were obtained at 25.9 °C under rough vacuum. FTIR spectra were normalized by dividing the absorbance intensity at each wavenumber by the absorbance intensity related to the ν_3_ band of the calcite located at 1400 cm^–1^. More details about the FTIR spectra normalization can be found in ref (21 and 30). ATR-FTIR spectral deconvolution was done by using 5 up to 9 Gaussian-like profiles.

XPS measurements were performed by using a monochromatic Al K_α_ source (XRM-50) and a NAP-PHOIBO analyzer. The surface charging was minimized by optimizing the energy and intensity of the electron beam (voltage and current: *I* = 50 μA, *V* = 3–10 V) of the flood gun. The sample preparation was made in air, and although minimal, adventitious carbon was expected at the surface. The binding energy referencing is done using Ca 2p_3/2_ core level (346.6 eV) due to its narrow line width. The spectral resolution was set at 0.5 eV for high-resolution spectra as measured on reference Ag sample at 3d_3/2_ core level peak with a pass energy 15 eV. To identify the binding energy position of ions of the LS water, XPS spectra of each dry salt (NaCl, CaCl_2_· 2H_2_O, MgCl_2_·6H_2_O, KCl, NaHCO_3_, and Na_2_SO_4_) were measured as a reference, as well as CaMg(CO_3_)_2_. C 1s core level was used to align the binding energy of the XPS spectra from salts. The XPS survey and the high-resolution spectra (C 1s, O 1s, and Ca 2p) were acquired for each sample. The survey was used to identify the elements at the surface: from the substrate (C, Ca, and O), from the oil (C), and from the brines (Mg, Ca, Na, S, and Cl). Element concentration was quantified after a Shirley background subtraction and accounting for the photoemission cross-section and the inelastic mean free path (IMFP). The C 1s core level spectra were fitted using up to six components reproduced using a GL function (product of Gaussian and Lorentzian line shape). The C 1s components were identified as carbonate, C_CO3_, adventitious C_adv_, water enhanced C_w_, oil C_oil_, aldehyde C_ald_, and hydrogenated CO_3_, i.e., HCO_3_, as in refs (21 and 30). XPS data analysis was performed using CASAXPS software.^31^

## Results and Discussion

### Calcite/LS: FTIR and XPS

**Figure 2:**
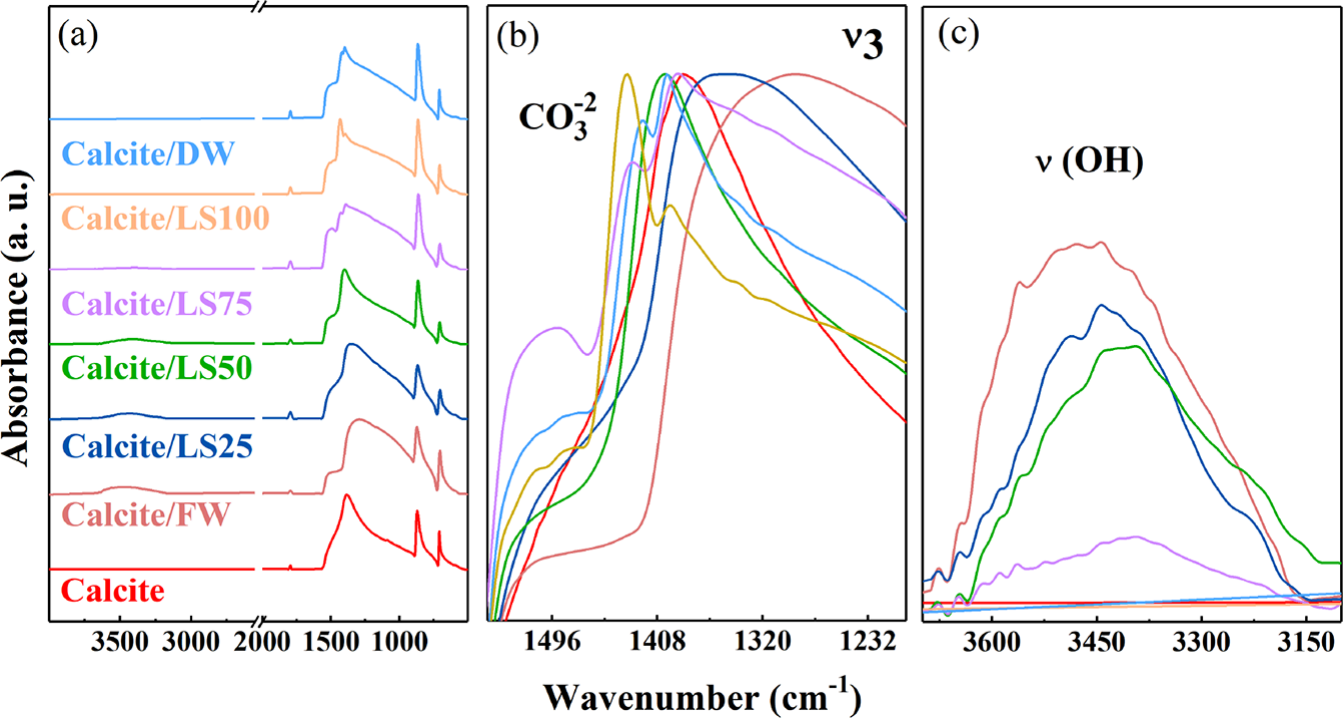
ATR-FTIR spectra of calcite single crystals conditioned in FW, DW, and LS waters (LS25, LS50, LS75, and L100) in the regions (a) 4000–400 cm^–1^ (overall spectra), (b) 1500–1200 cm^–1^ (ν_3_-vibration band of CO_3_^2–^), and (c) 3800–3000 cm^–1^ (OH-vibration band).

**Figure 3:**
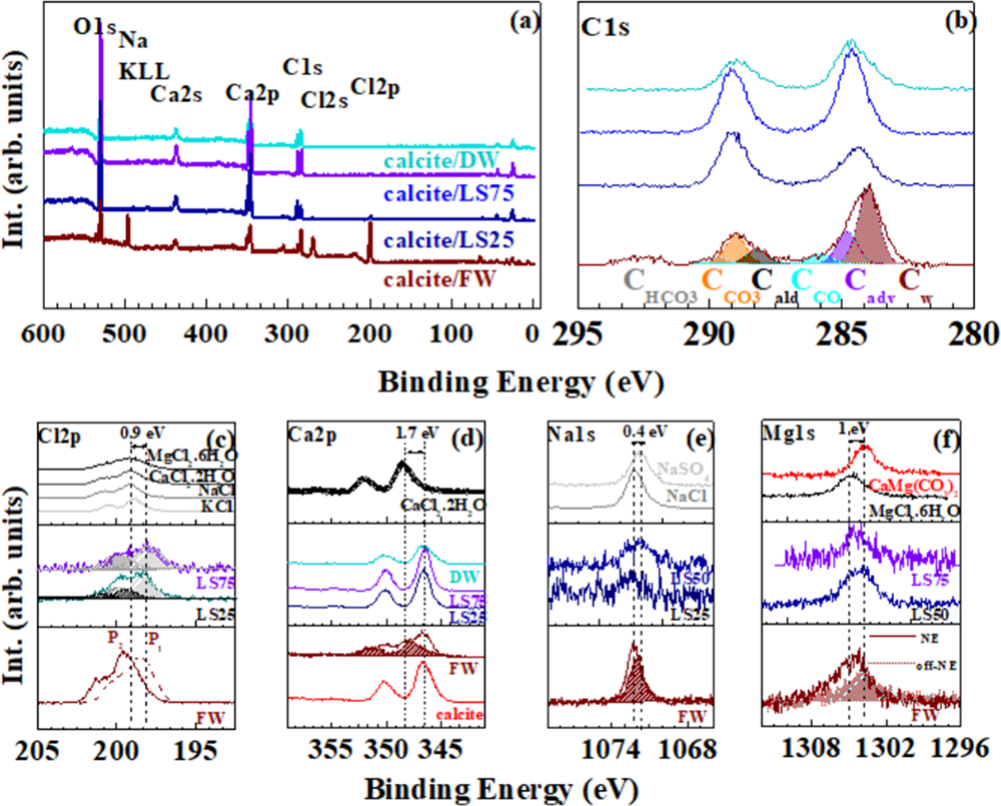
(a) XPS wide scan of calcites conditioned in FW (wine), LS 25 (navy blue), LS50 (blue), LS 75 (violet), and DW (dark cyan). (b) C 1s core level spectra of calcites conditioned with brines. Each component of the C 1s spectral deconvolution is shown for the calcite/FW surface. High-resolution XPS spectra in the binding energy region of Cl 2p (c), Ca 2p (d), Na 1s (e), and Mg 1s (f) core levels measured for reference salts (NaCl, CaCl_2_· 2H_2_O, MgCl_2_·6H_2_O, KCl, KCl, and Na_2_SO_4_) and calcite conditioned in brines in the same color code of panels (a) and (b). In the case of calcite conditioned with FW, the spectra measured at normal emission and 30° deg off normal emission are displayed.

The vibration mode of fresh, newly cleaved calcite may originate from CO_3_^2–^ (2000–700 cm^–1^) or calcium-related bands (<500 cm^–1^).^32,33^ Only the vibrations corresponding to the carbonate group are visible in Figure 2 and are identified at 1793 cm^–1^ (symmetric stretching and bending in the C–O plane, ν_1_ + ν_4_), 1411 cm^–1^ (asymmetric stretching, ν_3_), 867 cm^–1^ (out-of-plane curvature, ν_2_), and 709 cm^–1^ (bending in the C–O plane, ν_4_).^33^ The quality of the calcite cleavage was confirmed by the absence of the amorphous phase (split of the band at 1400 cm^–1^) and other carbonaceous polymorphs (vibration modes at 1475 and 856 cm^–1^ characteristic of aragonite).^34,35^ Upon LS condition with salinity lower than 3173 mg/L (i.e., LS75, LS100, and DW), the ATR-FTIR spectra showed ν_3_ band split in the wavenumber region 1550–1200 cm^–1^ (Figure 2b), while above this salinity, the ν_3_ band intensity increases and shifts toward lower wavenumbers up to 84 cm^–1^ for FW conditioning. Moreover, further vibration bands appear in the interval of 4000–3000 cm^–1^, attributed to ν_s_(OH) and O–H stretching, with intensity increasing with increasing salinity (Figure 2c). The alterations in the ν_3_ band may reflect the modification of the carbonate group environment: mixture of crystalline and amorphous (ν_3_ band split)^34,36^ promoted by the low concentrations of ions through the dissolution process and ion pair formation (ν_3_ shift) enhanced by waters with high concentrations of salts.^37,38^ The lack of ν_s_(OH) band in calcite/DW and calcite/LS100 can be concomitant with the competitive dissolution and readsorption processes, whereas most intense ν_s_(OH) bands in calcite/FW may reflect either a full hydrolyzed surface (O 1s core level spectra of fresh cleaved calcite^21^) or salt deposit formation (Figure 3). Moreover, ν_s_(OH) bands of calcite/FW differ in line shape from that of FW in the liquid phase (Figure 1S in SI), suggesting distinct OH environment formation at the surface.

Further insight about calcite/brine interfaces was obtained by the analysis of pristine and treated calcite surfaces using XPS. Figure 3 shows the XPS survey spectra of calcite conditioned with FW, DW, and some of the LS waters; the spectral features of elements from the substrate (Ca, C, and O) and saline ions (Na^+^, Cl^–^, Mg^2+^, and Ca^2+^) are highlighted. On its surface (104), calcite has dangling bonds originating from subcoordinated Ca and CO_3_^2^–^^ turning the surface chemically reactive. The extent of the interaction between the brines and calcite can be derived from the changes of the O/Ca, O/CO_3_, and Ca/CO_3_ ratios (extracted from the XPS intensity) with respect to the expected stoichiometric values: 3:3:1 (Table 1). Upon brine conditioning, subcoordinated Ca atoms are saturated by the OH group of water as testified by an invariant O/Ca ratio equal to 3 independent of the salinity. Moreover, the relative intensity of CO_3_^2^–^^ feature and O/CO_3_ and Ca/CO_3_ ratios for calcite/LS75 and calcite/DW interfaces was greater than that of the pristine surface, probably associated with the dissolution process.^39−41^ The calcite treated with LS waters with greater salinity (LS50, LS25) still presents O/CO_3_ and Ca/CO_3_ ratios that differ from that of pristine calcite, but in this case, either salt deposit or additional ion layer formation on the calcite surface cannot be excluded in the data interpretation. XPS survey spectra of the calcite/brines interface display a variation of Na, Cl, Mg, and Ca spectral feature intensity (Figure 3); it decreases by reducing the water salinity. Each element concentration was extracted from the XPS survey spectra and is reported in Table 1. The Cl signature is always detected within XPS sensitivity, but Na and Mg only for calcite treated with LS25, LS50 waters. Moreover, the C 1s core level spectra of the calcite/brine interface can be deconvoluted into several components associated with C_CO3_, C_adv_, C_w_, C_oil_, C_ald_, and C_HCO3_ species in agreement with previous reports.^21,30^ An additional component C_CO_ could be identified in the spectra.^41^ Upon brine conditioning, the spectral intensity of the C 1s core level was redistributed from mainly C_CO3_ (81%) to mostly C_w_, C_ald_, and C_CO_. To understand the alteration of calcite surface upon LS water treatment, the high-resolution XPS spectra in the binding energy region of Cl 2p, Ca 2p, Na 1s, and Mg 1s (Figure 3c–f) were acquired for each brine (FW, LS25, LS50, and LS75) and compared with the reference spectra measured for salts and CaMg(CO_3_)_2_. The Cl 2p core level spectra of the reference salts (NaCl, CaCl_2_·2H_2_O, MgCl_2_·6H_2_O, and KCl) show a similar binding energy position of ∼198.8(2) eV and a line width varying from 1.2 eV for KCl to 1.8 eV for MgCl_2_·6H_2_O. The binding energy position of Cl 2p_3/2_ for calcite treated with LS75 dislocates by 0.9 eV toward lower binding energy with respect to that of salts and is attributed to Cl^−^ adsorbed on the calcite surface. As far as the salinity increases, the Cl 2p spectral line shape is wider and the Cl 2p_3/2_/Cl 2p_1/2_ ratio is greater than the theoretical 0.5, i.e., a signature of the superposition of two contributions located at the binding energy of the adsorbed species (gray) and the salt (black). The presence of a black component may suggest that at this salinity, some of the Cl ions can be in the form of a deposit since the Cl environment is salt-like. At the calcite/FW interface, the prevalent contribution is that of a deposit-like, although the relative contribution may vary as a function of the position (the spectra are referred to two different points of the surface). As in the case of Cl^−^ ions, Na^+^ ions are also found in the XPS spectra of calcite/brine interface with a concentration that increases with salinity and a line shape compatible with two distinct contributions in calcite/FW: Na adsorbed on the surface and within the salt deposit. Unlike the Cl spectral feature, the binding energy dislocation of the Na 1s core level peak is toward higher binding energy by around 0.4 eV. The Mg 1s signal was only slightly above the background noise level and detectable for salinity above LS75. As in the case of the other ions, two Mg species could be distinguished at the calcite surface after the treatment: one at 1304.8(2) eV and the other dislocated by 1 eV toward lower binding energy. The first component is relative to the Mg still within the salt matrix, and the second has a binding energy compatible with Mg in CaMg(CO_3_)_2_. This means that even at room temperature, the formation of a superficial layer is possible where Mg is included in the calcite while interacting with brines. This is consistent with previous studies, where it was demonstrated that dolomite formation in natural systems is highly dependent on the geochemical environment, including Mg/Ca ratio, salinity, and pH.^42,43^ The Ca 2p_3/2_ binding energy position of the Ca ions within the CaCO_3_ and CaCl_2_· 2H_2_O salt occurs at 346.6 and ∼348.3 eV, respectively. No detectable changes in Ca 2p line shape could be observed in the calcite/LS interface, but in calcite/FW, an additional component in the spectra appears at the binding energy characteristic for CaCl_2_·2H_2_O.

**Table 1:** XPS Surface Elemental Concentration of Freshly Cleaved, Brine-Wet Calcite as Obtained from the XPS Spectra, i.e., Survey and C 1s Core Level

		calcite conditioned with				
samples	fresh cleaved calcite	DW	LS75	LS50	LS25	FW
ratio	survey					
O/Ca	4	3	3	3	3	3
O/C_CO3_	3	7	5	2.7	5	4
Ca/C_CO3_	0.75	2	2	1	1	3
conc%	survey					
Cl			1	1	1	22
Na				1	3	3
Mg						3
conc%	C 1s core level					
C_CO3_	81	39	39	23	26	21
C_w_	8	30	34	38	26	57
C_HCO3_	11	3	7	13	17	5
C_ald_		21	17	20	24	10
C_CO_		8	4	6	7	7

Both XPS and FTIR results indicate that calcite undergoes dissolution when treated with DW and very diluted brines (LS100). DW is ion-free, and it interacts with calcite, altering its surface relative chemical composition, in particular the CO_3_ environment. On the one hand, the quantitative analysis of XPS spectra provides the chemical composition of each brine-wet calcite in terms of Ca and CO_3_. On the other hand, the alteration ν_3_ vibration band was used to determine the extent of the dissolution process in brine-wet calcites.^34,35^ In the analysis of ATR-FTIR spectra, the prewet calcite with FW displays OH-vibration modes compatible with distinct water bonds. Moreover, the analysis of the similarly treated calcite surface clearly showed signatures of Na, Cl, Ca, and Mg elements in the XPS spectra; they could be found in different chemical environments: adsorbed at the surface and trapped within the salt deposit. Therefore, both FTIR and XPS indicate that the surface of prewet calcite with FW presents a different distribution of superficial elements and, therefore, other favorable adsorption sites for Nujol.

### Calcite/Nujol, Calcite/FW/Nujol, and Calcite/DW/Nujol

**Figure 4:**
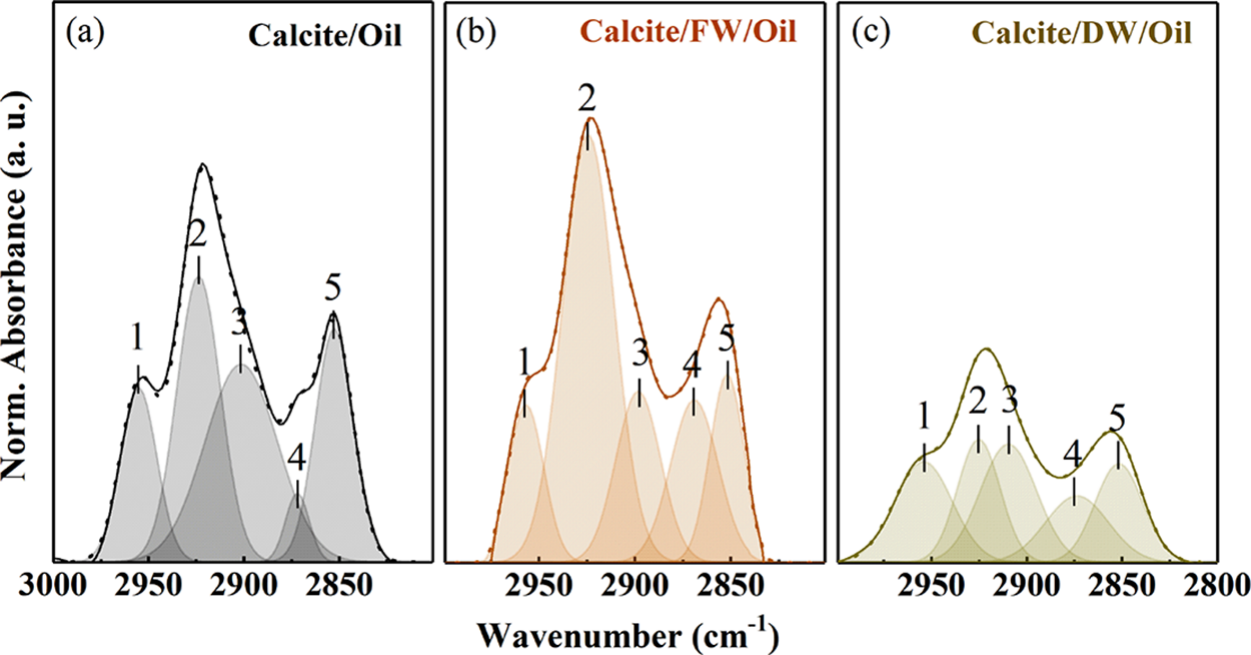
ATR-FTIR spectra of Nujol vibration band in the wavenumber region of 3000–2800 cm^–1^ and their deconvolution: (a) calcite/Nujol, (b) calcite/FW/Nujol, (c) calcite/DW/Nujol.

Figure 4 shows the ATR-FTIR spectra of (a) calcite/oil, (b) calcite/FW/Nujol, and (c) calcite/DW/Nujol in the interval 3000–2800 cm^–1^ with their deconvolution (table with the details of the fitting procedure for calcite/Nujol is reported in SI). An optimal deconvolution of the Nujol band profile of calcite/Nujol required five components identified as vibration modes [ν_ass_(CH_3_) at 2954 cm^–1^, ν_ass_(CH_2_) at 2923 cm^–1^, ν_sym_ CH_3_ at 2872 cm^–1^, and ν_sym_ CH_2_ at 2852 cm^–1^). The CH_2_ asymmetric stretching band (ν_ass_ CH_2_), assigned to 2923 cm^–1^, actually has a contribution from a hidden component at 2900 cm^–1^, identified as a Fermi resonance,^44^ generally present in alkyl chains with at least two nearly equivalent neighboring CH_2_ groups.^45^ The overall spectral intensity varies as a function of surface pretreatment, and it is maximum for calcite/FW/oil (Figure 4b), indicating that oil anchoring at the surface could be favored by the emergence of new adsorption points induced by FW hydration (water band). Moreover, ν_ass_(CH_3_) and ν_sym_(CH_3_) spectral features are blurred in the ATR-FTIR spectrum of calcite/FW/oil. These changes can be attributed to variation in the composition and structure of the calcite surface induced by the hydration process. The Nujol vibration band of calcite/DW/Nujol presents relative intensity redistribution within its components, such as CH_3_ and CH_2_ (Figure 4c).

### Calcite/FW/Oil/LS and Calcite/DW/Oil/LS

**Figure 5:**
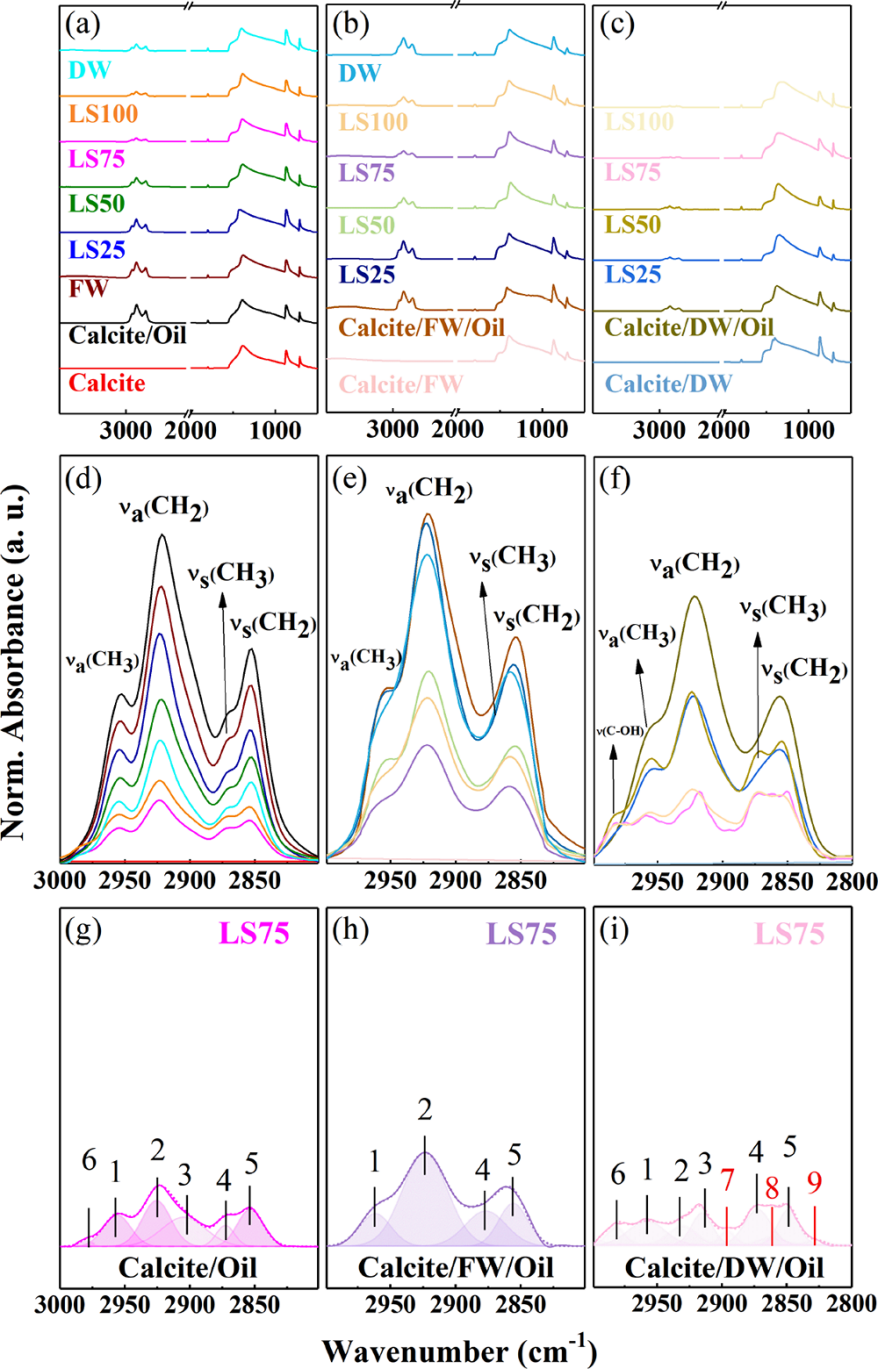
ATR-FTIR spectra of freshly cleaved calcite (a), prewet with FW (b), and DW (d) subsequently treated with Nujol and LS waters (LS25, LS50, LS75, and L100). The spectra are present in the absorption regions at (a–c) 3500–500 cm^–1^ (overall spectra) and (d–f) 3000–2800 cm^–1^ (CH_2_ and CH_3_ vibration bands). For selected specimens (g) calcite/oil/LS75, (h) calcite/FW/oil/LS75, and (i) calcite/DW/oil/LS75, the ATR-FTIR spectra deconvolution is illustrated in the region 3000–2800 cm^–1^.

Figure 5 shows the ATR-FTIR spectra in the relevant spectral ranges 3500–500 and 3000–2800 cm^–1^ of pristine (a–d)^21^ and hydrated calcites with FW (b–e) and DW (c–f), followed by conditioning in Nujol and subsequently in LS25 (navy blue), LS50 (light green), LS75 (lilac), LS100 (orange), and DW (light blue). Calcite surfaces that underwent treatment according to sequences 2, 3, and 4 do not show anymore the ν_3_ band spectral modification (ν_3_ band split for DW and shift for FW) of the carbonate (Figure 5c,d) probably due to the subsequent surface reorganization with Nujol molecules. In the case of calcite prior hydrated with FW, the vibration band was ∼1500 cm^–1^ (see Figure 3S in SI) associated with the COO– stretching modifies over the step of sequence 3: (i) it is absent in freshly cleaved calcite, (ii) appears after FW conditioning, (iii) decrease in intensity after Nujol wetting, and (iv) it is enhanced by further LS treatment. The Nujol vibration band intensity quantifies the oil present at the calcite surface after the last treatment of the sequence, and it varies with LS water salinity: (i) gradually in calcite/oil/LS and reaching the minimum with LS75 and increasing again for LS100 and DW (Figure 5d); (ii) more abruptly for calcite/FW/oil/LS and calcite/DW/oil/LS where the intensity decreases for salinity less than 4760 (mg/L), i.e., LS50, and being minimum for LS75. In the case of calcite/DW/oil/LS the last treatment with LS75 and LS100 shows the minimum oil band (i.e., the greater oil removal) and significant line shape changes (Figure 5f) with respect to the calcite without prior hydration.

Figure 5g–i displays the spectral deconvolution of calcite prior nonhydrated and hydrated and conditioned with Nujol and LS75. In the case of nonhydrated calcite (Figure 5g), a new component appears at ∼2990 cm^–1^ (band 6), identified as C–OH stretching (ν(C–OH)) and/or =C–H stretching (ν(=C–H)), not observed before in the liquid Nujol (Figure 2S in SI) and the conditions with the highest salt content.^21,46−50^ Calcite/FW/oil/LS75 presents an overall decrease of Nujol band spectral intensity as shown in Figure 5h but not significant spectral line shape variation with respect to Figure 4b. Band 3 (Fermi resonance) was not observed under these conditions, which may suggest that during the hydration process, the neighboring CH_2_ groups lose their equivalence upon adsorption since this resonance typically arises from the presence of nearly equivalent adjacent CH_2_ groups. The spectral line shape of Nujol band in calcite/DW/oil/LS75 is significantly modified with well-defined additional peaks. Three new components are required to properly reproduce the spectral line and centered at 2897 cm^–1^ (band 7) =CH–R vibration, 2865 cm^–1^ (band 8) O–CH_3_ vibration, and 2828 cm^–1^ (band 9) □–H (Figure 5i).^51,52^

The =CH–R vibration corresponds to the asymmetric stretching of the C–H bonds in alkyl groups (R), which can arise as a result of the interaction between the Nujol and the calcite surface. Nujol may contain a small amount of C=C bonds in its chain, and these bonds may change after the interaction with LS and give rise to this vibration. The O–CH_3_ vibration is attributed to stretching of the C–H bonds in methoxy groups suggesting that the oxygen on the surface of the calcite is interacting with the oil molecules. The band at ∼2828 cm^–1^ (□–H) is the result of the bond between an H and an oxygen defect. In fact, similar results were observed for hydrogen bonded to oxygen defects in diamond structures after its dissolution.^47^ These vibrations highlight the complexity of interactions on the dissolved surface, showing specific Nujol absorption depending on the elemental composition and surface conformation.

**Figure 6:**
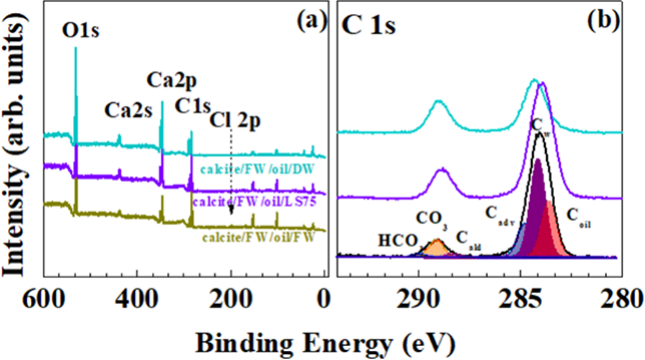
XPS survey (a) and C 1s high-resolution core level (b) spectra performed on the calcite/FW/oil surface conditioned with DW (dark cyan), LS75 (violet), and FW (dark yellow). The main core level photoemitted electrons are highlighted for each element in the survey. The representative deconvolution of the C 1s core level spectrum is highlighted for the calcite/FW/oil/FW specimen: C_CO3_, C_adv_, C_w_, C_oil_, C_ald_, and C_HCO3_.

To identify the role of prior hydration on the oil removal from calcite surfaces, the sequence of calcite/FW/oil/LS was investigated by XPS. Figure 6 shows the XPS survey and the C 1s core level of calcite/FW/oil/FW (dark yellow), calcite/FW/oil/LS75 (violet), and calcite/FW/oil/DW (dark cyan). The conditioning with brines of the calcite/FW/oil results in an increase of O/Ca and O/C_CO3_ ratios with respect to the pristine and only hydrated surface but an almost invariant Ca/C_CO3_ close to 1; all surfaces present oil (C_oil_) left over them, with the greater content on the one treated with FW (Table 2). The conditioned prehydrated calcite surfaces show an intensity reduction of features related to Ca and CO_3_, probably due to the oil and water layers left over them, whereas the increase in the content of the O may be related to the OH group bond either directly to the substrate or over the fluid layers. Unlike hydrated surfaces, where Cl^–^ ions and traces of Na^+^ and Mg^2+^ [surface either bonded to the surface or in salt deposits (Figure 3)] could also be identified at the calcite surface, on calcite/FW/oil/brines, only Cl element was detected. The presence of specific ions on the surfaces of calcite/brines and calcite/FW/oil/brines can be explained by Mg^2+^, Ca^2+^, Cl^–^, and Na^+^ mobility ^+^. In fact, Mg^2+^ has the greatest mobility in solution and may interact with the calcite matrix, inducing its incorporation at the surface.^25,26^ However, in calcite/FW/oil/brines, only Cl^–^ was observed and the absence of the Mg^2+^, Ca^2+^, and Na^+^ ions suggest their active role in the oil removal process.

**Table 2:** XPS Surface Elemental Concentration of Freshly Cleaved, Calcite/FW/Oil/DW, Calcite/FW/Oil/LS75, and Calcite/FW/Oil/FW as Obtained from the XPS Spectra, i.e., Survey and C 1s Core Level

	calcite/FW/Oil conditioned with			
samples	fresh cleaved calcite	DW	LS75	FW
ratio	survey			
O/Ca	4	5	9	7
O/C_CO3_	3	5	7	10
Ca/C_CO3_	0.75	1	0.8	1.3
conc%	survey			
Cl			1	2
Na				
Mg				
conc%	C 1s core level			
C_CO3_	81	26	20	10
C_w_	8	47	64	55
C_oil_		9	13	31
C_HCO3_	11	12	1	2
C_ald_		6	3	2

### Mechanism

In a previous work, Nujol, directly in contact with the calcite surface, forms a film weakly bonded with the substrate.^21^ The wettability alteration toward a water-wet condition was demonstrated by the greatest reduction of the Nujol vibration mode intensity after treatment with the LS water with salinity as high as 3173 mg/L (81% of initial content) and also confirmed by the minimum contact angle (17°). In this condition, the Nujol continuous film is reduced to a fragmented island on the calcite surface. Moreover, the oil removal efficiency was not improved by enhancing Ca^2+^ and Mg^2+^ contents in the brine, indicating that the presence of both ions (Ca^2+^ and Mg^2+^) is important, although the Mg^2+^ ion has a pivotal role in the oil removal effect. Cl^–^ and Na^+^ left over the calcite/Nujol surface after conditioning with brines indicates that they have enough high mobility through oil to reach the calcite surface, with the more inert Cl^–^ kept on it and the other either participating in the oil removal process or farther from the surface.^30^

**Figure 7:**
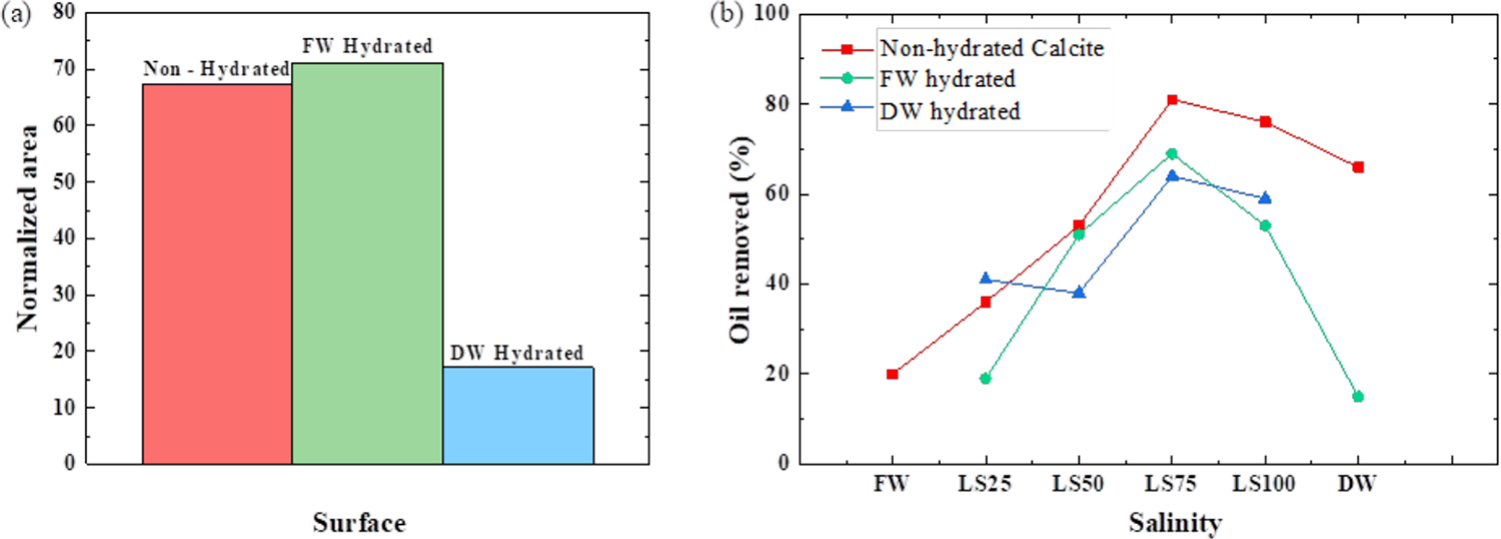
(a) Normalized area of the vibration band of Nujol extracted from the FTIR spectra for nonhydrated (red) and prior hydrated calcite with FW (green) and DW (light blue). (b) Removed oil from the calcite surface upon conditioning with LS nonhydrated (red) and prior hydrated calcite with FW (green) and DW (light blue).

In calcite wetted by FW, the OH group was detected in correspondence of the ν_*s*_(OH) band associated with either group within water or hydroxylated surface; and CO_3_ surface atom coordination modification occurs as testified by the shift toward lower wavenumber by 84 cm^–1^ of the ν_3_ vibration band of calcite. Some of the ions (Cl^–^, Na^+^, and Mg^2+^) left over from the wetting process on the calcite are bonded with its surface, and others are found with a salt deposit (Figure 3). In particular, detectable Mg ions form a layer of CaMg(CO_3_)_2_ over the calcite surface. Conversely, the split of the ν_3_ vibration band of calcite conditioned with DW is the signature of its surface crystalline alteration. The calcite prehydration modifies its surface composition and how Nujol bonds to it. The oil amount adsorbed on the calcite surfaces was greatest on the one prior hydrated with FW and lowest on the one hydrated with DW, a behavior that can be explained through the different states of the calcite surface: ion adsorption (FW) vs dissolution (DW) (Figure 7a). Further conditioning with oil and LS water of prewet calcite with FW or DW (sequences 3 and 4) was used to investigate the oil removal process from the calcite surface. In all conditions, the intermediate salinity brine (LS75) showed the best oil removal; possibly, the active ions (Mg^2+^, Ca^2+^, and SO_4_^2–^) cross the oil film in a more effective way than the less active ones (Cl^–^ and Na^+^) due to surface charge exchange (Figure 7b).

## Conclusions

Specific sequences of calcite preparation (prehydration with either FW or DW) were undertaken to understand the oil removal mechanism by reproducing conditions closer to those of an oil reservoir, where both oil and brines can simultaneously be present on the calcite surface. A preliminary characterization of calcite upon LS water conditioning indicates that Ca interacts with the OH group and retains it at the surface, and CO_3_^2–^ undergoes crystalline modification (shift and split) attributed to new bonds (FW) or dissolution (DW). Cl^–^, Na^+^, and Mg^2+^ are clearly identified at the calcite/FW surface, either bonded with the substrate matrix or within a salt deposit. Combined FTIR and XPS results reveal an altered calcite surface upon brine treatment. The change in the termination of the calcite surface resulted in different oil anchoring on the surface; more oil is adsorbed on calcite/FW with respect to pristine and calcite/DW. Despite the different calcite termination, after the last treatment, the optimal LS water for the oil removal process was the same for every calcite surface (pristine and prehydrated), the one with salinity 3173 mg/L and corresponding to LS75. This further indicates that the LS ion strength is more relevant for the calcite surface termination.

## Supporting Information Available

Comparison between FTIR spectra of liquid FW and FW/calcite; FTIR spectra of liquid Nujol; FTIR spectra of LS/oil/FW/calcite in the wavenumber region of 1500 cm^–1^; and explanation of the deconvolution of the Nujol FTIR spectra (PDF)
