# Supplementary material for: Oil Removal in Prewet Calcite: Active Versus Inactive Ions Investigated by a Fourier Transform Infrared and X-ray Photoelectron Spectroscopy Study
Source: Langmuir. 2025 Apr 7;41(15):9848–56. doi: 10.1021/acs.langmuir.5c00286 (PMC12020411; doi:10.1021/acs.langmuir.5c00286)
Supplement: Supplementary file 1 — la5c00286_si_001.pdf [file la5c00286_si_001.pdf]

Oil removal in pre-wet calcite: The actives vs inactive ions. A Fourier Transform Infrared and X-ray photoeletron spectroscopy study

J.M. Loreto[1,3], E. Annese[1,4], L. G. Pedroni[3], and F. Stavale[1],

[1] Brazilian Center for Research in Physics (CBPF), Rio de Janeiro – Brazil

[2]Center "Leopoldo Américo Miguez de Mello" for Research and Development (CENPES), Petrobras S.A., Rio de Janeiro – Brazil

[3] Chemistry Department, Pontifical Catholic University of Rio de Janeiro, Rio de Janeiro – Brazil

[4]Instituto de Física, Universidade do Estado do Rio de Janeiro UERJ, Rua São Francisco

Xavier, 524, Rio de Janeiro, RJ 20550-013, Brazil

1. FTIR of liquid FW and FW/calcite

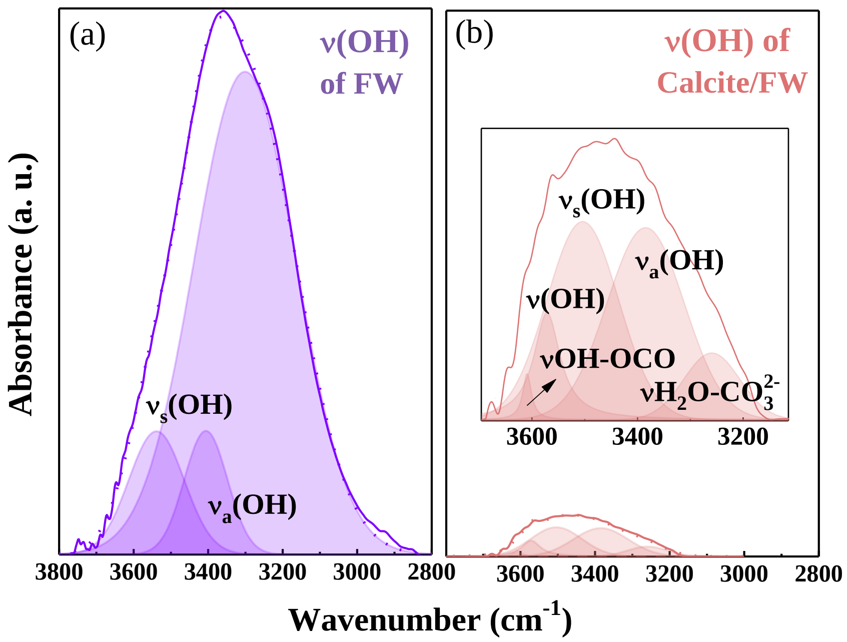

Figure 1S - ATR-FTIR spectra in the region 3800 – 2800 cm^{-1} of (a) Formation water (b) Calcite conditioned with FW.

The FTIR spectra of in the region 3000 cm^{-1} – 2800 cm^{-1} of the OH vibrations of liquid FW is compared with the calcite aged in FW in **Error! Reference source not found.**S. The the ν(OH)) band of liquid FW in Figure 1S (a) was deconvoluted into three Gaussians centred: at ~3538 cm^{-1} (symmetric stretching, νs(OH)), ~3405 cm^{-1} (asymmetric stretching, νa(OH)), and at ~3300 cm^{-1} ("network", the water molecules strongly bonded to hydrogen) [1, 2, 3]. The ν(OH)) band of calcite conditioned with FW in Figure 1S (b) was deconvoluted into 6 bands centred at: ~3648 cm^{-1} (νOH-OCO), 3609 cm^{-1} (isolated OH, ν(OH)), ~3538 cm^{-1} (νs(OH)) and ~3403 cm^{-1} (νa(OH)) of water, at ~3384 cm^{-1} (OH vibrations of non-hydrogen-bonded water molecules) and at ~3238 cm^{-1} (stretching mode of the interaction between carbonate anions and water molecules, located in the interlayers, νH_2O -CO_3^{2-}) [4,5,6].

2. Spectrum of Nujol liquid

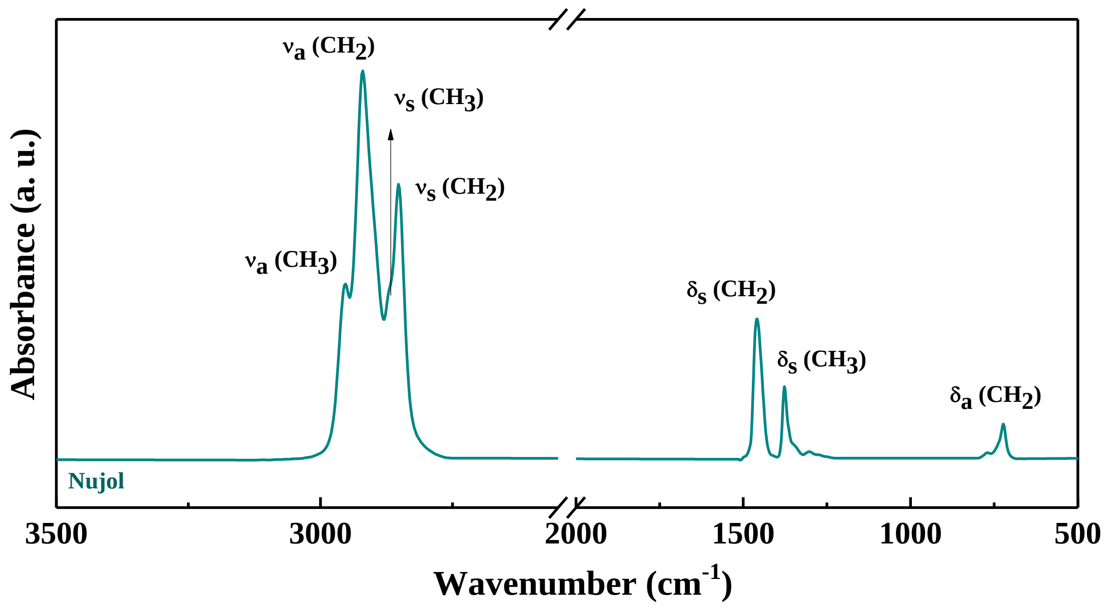

Figure 2S - ATR-FTIR Spectrum of Nujol liquid in the region 3500 – 500 cm^{-1}. The Nujol vibrational bands are identified and highlighted: $\nu_a(CH_3)$ (asymmetric stretching CH_3), $\nu_a(CH_2)$ (asymmetric stretching CH_2), ν_s (CH_3) (symmetric stretching CH_3), ν_s (CH_2) (symmetric stretching CH_2), δs (CH_2) (symmetric deformation (scissors) CH_2), δs (CH_3) (symmetric deformation (umbrella) CH_3) and δs (CH_2) (asymmetric deformation (rocking) CH_2).

Figure 2S displays the Nujol FTIR spectrum characterized by the typical bands of linear hydrocarbons, associated with the asymmetric stretching CH_3 ($\nu_a(CH_3)$) at 2954 cm^{-1}, asymmetric stretching CH_2 ($\nu_a(CH_2)$) at 2923 cm^{-1}, symmetric stretching CH_3 (ν_s (CH_3)) at 2872 cm^{-1} and symmetric stretching CH_2 (ν_s (CH_2)) em 2852 cm^{-1}. The region at 1500 cm^{-1} – 500 cm^{-1} which is characterized by the bands in the region between 1480-1450 cm^{-1} and 1380-1350 cm^{-1} related to symmetric (scissor) deformation CH_2 (δs (CH_2)) and symmetric (umbrella) deformation CH_3 (δs (CH_3)), respectively. The absence of long, thin bands in the region between 1500 cm^{-1} e 1600 cm^{-1}, characteristic of aromatic compounds, confirms the linear nature of the compound. The band observed at approximately 720 cm^{-1} is identified as asymmetric deformation (rocking) of the CH_2 group CH_2 (δs (CH_2)), ypical for long-chain alkenes [7,8,9,10,11].

3. LS/oil/FW/calcite

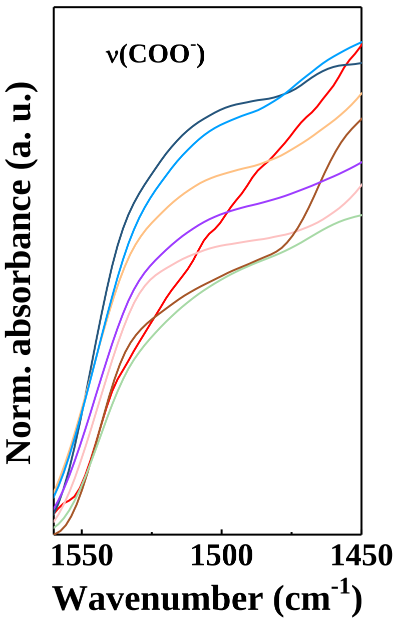

Figure 3S - ATR-FTIR spectra of pre-wet calcite with FW and subsequently treated with Nujol and brines in the interval: 1550 – 1450 cm^{-1}.

The vibrational band at ~1500 cm^{-1} in Figure 3S corresponds to the COO- stretching absent in freshly cleaved calcite and manifests as a result of surface modification upon interaction with FW. Its intensity decrease after conditioning in nujol and it is enhanced by further LS treatment: a behavior that can be explained by a modification of COO- functional group not anymore active due to oil adsorption and again available due to the action of the brines.

4. Deconvolution Oil bands

All the deconvolutions were carried out using Gaussians in the Fityk software, which is common for FTIR spectra [12,13,14]. The number and position of the bands were determined by the minimum in the second derivative spectra in the region of interest [15]. An example is shown in Figure 4S, which shows the 3000 – 2800 cm^{-1} vibration region of FTIR spectra of calcite with oil and its constituents. The minimum of each peak in the second derivative (in red) showed the location of the bands in this region. The parameters of the fits for this region are shown in Table 1S and are close to the values reported for the same spectral range in coal samples [16,17]. We kept the band centers fixed and the other parameters of the fits free, in order to identify shifts and variations in the linewidth, which may show changes in the chemical environment and specific molecular interactions, as observed in similar studies [15,18]. The parameters were fitted to the experimental data using

an interactive least squares procedure, and the result was evaluated both visually and by statistical methods, ensuring the best possible curve fit.

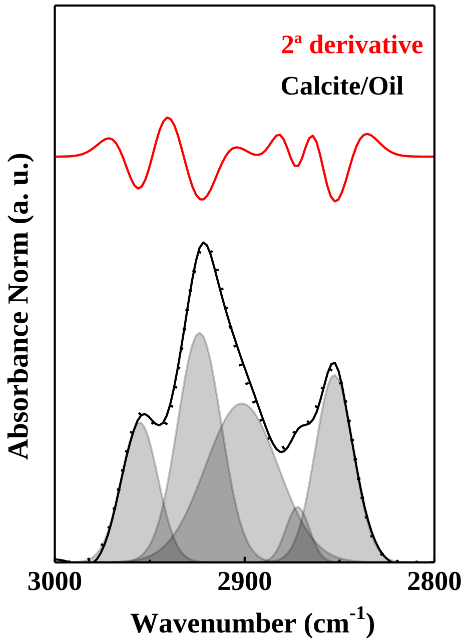

Figure 4S - FTIR spectrum of calcite with oil, showing five overlapping bands that appear as three main bands and a shoulder in the original FTIR spectrum. The second derivative (in red) shows the location of each band. The curves were fitted using Gaussian profiles, highlighting the bands that had been masked in the spectrum.

Table 1S - Deconvolution parameters for the 3000 to 2800 cm-1 region for calcite conditioned in oil, obtained from curve fitting using the Fityk software.

Band center	FWHM	Area	Intensity
2995	22,6	7,8	0,3
2923	27,0	15,5	0,5
2900	45,0	17,8	0,4
2872	19,6	2,0	0,1
2852	23,5	10,9	0,4

References

[1] Onori, G., & Santucci, A. (1993). IR investigations of water structure in Aerosol OT reverse micellar aggregates. The Journal of Physical Chemistry, 97(20), 5430-5434.
[2] Boissiere, C., Brubach, J. B., Mermet, A., De Marzi, G., Bourgaux, C., Prouzet, E., & Roy, P. (2002). Water confined in lamellar structures of AOT surfactants: an infrared investigation. The Journal of Physical Chemistry B, 106(5), 1032-1035.
[3] Laurson, P., Raudsepp, P., Kaldmäe, H., Kikas, A., & Mäeorg, U. (2020). The deconvolution of FTIR-ATR spectra to five Gaussians for detection of small changes in plant–water clusters. AIP Advances, 10(8).
[4] Drenchev, N. L., Chakarova, K. K., Lagunov, O. V., Mihaylov, M. Y., Ivanova, E. Z., Strauss, I., & Hadjiivanov, K. I. (2020). In situ FTIR spectroscopy as a tool for investigation of gas/solid interaction: Water-enhanced CO2 adsorption in UiO-66 metal-organic framework. JoVE (Journal of Visualized Experiments), (156), e60285.
[5] Walrafen, G. E. (1972). Water: a comprehensive treatise. by F. Franks, Prenum Press, New York, 1, 151.
[6] Bharmoria, P., Gupta, H., Mohandas, V. P., Ghosh, P. K., & Kumar, A. (2012). Temperature invariance of NaCl solubility in water: Inferences from salt–water cluster behavior of NaCl, KCl, and NH4Cl. The Journal of Physical Chemistry B, 116(38), 11712-11719.
[7] Dong, L., Jiao, F., Qin, W., & Liu, W. (2019). Selective flotation of scheelite from calcite using xanthan gum as depressant. Minerals Engineering, 138, 14-23.
[8] Cui, Y., Jiao, F., Wei, Q., Wang, X., & Dong, L. (2020). Flotation separation of fluorite from calcite using sulfonated lignite as depressant. Separation and Purification Technology, 242, 116698.
[9] Hill, I. R., & Levin, I. W. (1979). Vibrational spectra and carbon–hydrogen stretching mode assignments for a series of n-alkyl carboxylic acids. The Journal of Chemical Physics, 70(2), 842-851.
[10] Roy, d., das, n. M., & gupta, p. (2012). Study of cadmium arachidate multilayers deposited by langmuir-blodgett technique. Materials science and engineering (ijmmse), 2(3), 1-10.
[11] Solomons, T. G., & Fryhle, C. B. (1999). Química orgânica (No. QD251. 2. S64 1979.). Limusa.
[12] Rahman, S. K., Rahma, A., Syauqiah, I., & Elma, M. (2020). Functionalization of hybrid organosilica based membranes for water desalination–Preparation using Ethyl Silicate 40 and P123. Materials Today: Proceedings, 31, 60-64.
[13] Ortuso, R. D., Ricardi, N., Bürgi, T., Wesolowski, T. A., & Sugihara, K. (2019). The deconvolution analysis of ATR-FTIR spectra of diacetylene during UV exposure. Spectrochimica Acta Part A: Molecular and Biomolecular Spectroscopy, 219, 23-32.
[14] Wojdyr, M. (2010). Fityk: a general-purpose peak fitting program. Journal of applied crystallography, 43(5), 1126-1128.
[15] Asemani, M., & Rabbani, A. R. (2020). Detailed FTIR spectroscopy characterization of crude oil extracted asphaltenes: Curve resolve of overlapping bands. Journal of Petroleum Science and Engineering, 185, 106618.
[16] Ibarra, J., Munoz, E., & Moliner, R. (1996). FTIR study of the evolution of coal structure during the coalification process. Organic geochemistry, 24(6-7), 725-735.
[17] Li, K., Khanna, R., Zhang, J., Barati, M., Liu, Z., Xu, T., ... & Sahajwalla, V. (2015). Comprehensive investigation of various structural features of bituminous coals using advanced analytical techniques. Energy & fuels, 29(11), 7178-7189.

[18] Wang, Q., Hua, Z., & Guan, J. (2019). Structure of Wangqing oil shale and mechanism of carbon monoxide release during its pyrolysis. Energy Science & Engineering, 7(6), 2398-2409.
